# Lung cancer In non-smokers

**DOI:** 10.1001/jama.2025.17695

**Published:** 2025-11-25

**Authors:** Cian Murphy, Tej Pandya, Charles Swanton, Benjamin J. Solomon

**Affiliations:** 1 Cancer Evolution and Genome Instability Laboratory, The Francis Crick Institute, London, UK; 2 The Royal Marsden Hospital, London, UK; 3 UKRI UCL Centre for Doctoral Training in AI-enabled Healthcare Systems, University College London, London, UK; 4 Cancer Research UK Lung Cancer Centre of Excellence, University College London Cancer Institute, London, UK; 5 Department of Oncology, University College London Hospitals, London, UK; 6 Department of Medical Oncology, Peter MacCallum Cancer Centre, Melbourne, Victoria 3000, Australia; 7 Sir Peter MacCallum Department of Oncology, University of Melbourne, Parkville, Victoria 3052, Australia

## Abstract

**Importance:**

Lung cancer in non-smokers (defined as people who have smoked fewer than 100 cigarettes in their lifetime) accounts for 15% to 25% of all lung cancer cases worldwide. In the US, the annual incidence of lung cancer in non-smokers is 14-20 per 100,000 person-years in women and 4-13 per 100,000 person-years in men.

**Observations:**

Most lung cancers in non-smokers (60-80%) are adenocarcinomas, compared with 40% among current or former smokers with lung cancer. The remainder are adenosquamous (10-20%), squamous (10-15%) and small cell lung cancer (0-5%). Risk factors include exposure to passive smoking (percentage attributable fraction: 15%), radon exposure (9%), air pollution (8%), asbestos (8%), and history of lung cancer in a first-degree family member (26%). Therapeutically targetable genomic variants such as *EGFR* variants or *ALK* gene rearrangements are more common in tumors from non smokers compared to those with a smoking history (43% vs 11% for *EGFR* and 12% vs 2% for *ALK*). In contrast, tumor mutation burden, the number of somatic mutations in a tumor cell, is lower in lung cancer among non-smokers (0-3 mutations per Megabase (Mb) vs 0-30 mutations/Mb). Similar to individuals with a history of smoking, non-smokers with lung cancer may present with wheeze, chest pain, dyspnea, hemoptysis or symptoms attributable to metastatic disease (eg, bone pain and headache) or be diagnosed with incidentally detected disease.The US Preventive Services Task Force (USPSTF) does not currently recommend lung cancer screening with low-dose CT scans for non-smokers, although screening guidelines vary globally.

Treatment typically involves a combination of surgery, radiotherapy and systemic therapies depending on stage, performance status and molecular features of the tumor. Comprehensive next-generation sequencing should be performed on Stage Ib to IIIa lung cancer tumor tissue from non-smokers because actionable genomic alterations such as *EGFR* variants or *ALK* gene rearrangements are treated with targeted therapy such as the tyrosine kinase inhibitors osimertinib or lorlatinib, respectively. Median survival among non-smokers with advanced NSCLC (Stage IIIb or higher) and actionable genomic alterations can exceed 3-5 years, while survival without these genomic alterations is similiar to lung cancer in smokers (1-2 years).

**Conclusions:**

Lung cancer in non-smokers accounts for 15-20% of lung cancer cases worldwide. Among patients with lung cancer, non-smokers are more likely to have genomic alterations such as *EGFR* variants or *ALK* gene rearrangements, and these patients have improved survival when treated with tyrosine kinase inhibitors, compared with chemotherapy.

## Introduction

Lung cancer is the leading cause of cancer-related mortality worldwide, causing approximately 1.8 million deaths in 2022^1^. Although tobacco smoking is the predominant risk factor for lung cancer, present in 80–85% of cases, smoking rates are declining in the US and in many other parts of the world. In parallel with a decrease in adult US smoking rates (23.3% in 2000 vs 11.5% in 2021^2^), lung cancer incidence in the US declined from 68/100,000 in 2000 to 47/100,000 in 2021^3^.

Lung cancer among non-smokers (individuals who have smoked fewer than 100 cigarettes in their lifetime) accounts for 15-25% of all lung cancer^4,5^, and may be related to factors including age, air pollution, passive smoking, radon exposure, asbestos exposure and germline genetic risk^6–8^. The misperception that lung cancer is almost invariably caused by smoking may delay assessment and diagnosis^9,10^. This review will discuss the epidemiology, risk factors, screening, clinical presentation, assessment, diagnosis, treatment and prognosis of lung cancer in non-smokers.

## Methods

A PubMed search for English-language articles describing studies on lung cancer in non-smokers was conducted from January 1, 2005 to August 1st, 2025. In total, 902 studies were retrieved from this search and 92 were included in this review, consisting of 6 meta-analyses or systematic reviews, 16 randomised clinical trials, 8 prospective cohort studies, 7 retrospective cohort studies, 3 cross-sectional studies, 4 observational or case-control studies, 13 genomic or molecular studies, 11 narrative reviews, 10 statistical or surveillance reports, 11 guidelines or recommendation statements, 2 registry or database studies, and 1 preclinical or experimental study.

### Epidemiology and Screening

Most lung cancers in non-smokers are adenocarcinomas (60-80%), and are diagnosed at a median age of 67 years, compared with age 70 years in former or current smokers (Table 1)^11^. The absolute incidence of lung cancer in non-smokers in the US and worldwide is increasing. A retrospective study of 10,000 cases from 3 US hospital networks reported that the proportion of lung cancer among non-smokers increased from 8% to 14.9% from 1990-2013^4^. A pooled analysis of 7 Finnish cohorts reported an absolute increase in lung cancer among non-smokers from 6.9 per 100,000 person-years in 1972 to 12.9 per 100,000 person-years in 2015^12^. Lung cancer is the third most diagnosed cancer worldwide, and if lung cancer in non-smokers were classified as a distinct entity, it would be the 7^th^ most common cancer^13^.

There are substantial ethnic, racial and geographic differences in the incidence of lung cancer in non-smokers. The age-adjusted incidence rate of lung cancer in non-smokers among female Asians in the US between 2000 and 2013 was 17.5 per 100,000 individuals (95% CI, 15.0-20.2) compared with 10.1 per 100,000 (95% CI 9.0-11.3) in non-Hispanic White females^14^.

The incidence of lung cancer in non-smokers worldwide is higher among women than men, however this may be confounded by the difference in the population at risk^15,16^. In Taiwan, up to 83% of non-smokers with lung cancer cases occur in women^5,17,18^. Smoking has historically has been higher among males than females and there are concerns about the accuracy of data on smoking status^18^. For example, a study of electronic healthcare records of 16,874 individuals in the US reported 80% had inaccuracies in their smoking history^19^. In addition, the US Surveillance, Epidemiology, and End Results (SEER) program, which aggregates survival data from lung cancers registered in the US, does not record smoking status^20^.

A pooled analysis of 7 Finnish cohorts demonstrated an age-standardized increase in lung cancer among female non-smokers (0.4 per 100,000 person-years in 1972 vs 6.2 per 100,000 person-years in 2015) compared with a stable rate of lung cancer among male non-smokers (6.5 per 100,000 person-years in 1972 vs 6.7 per 100,000 person years in 2015^12^. A study that included 6 population-based cohorts (n=1,364,658 individuals, n=5379 incident lung cancer case) reported that the age-adjusted incident rate ranged was 14.4-20.8 per 100,000 person-years in non-smoking females and 4.8 -12.7 per 100,000 person-years in non-smoking males^21^.

### Environmental Risk Factors

Several environmental exposures are associated with an increased risk of lung cancer in non-smokers (Table 1). Radon, a colorless gas that arises from the radioactive decay of uranium found in rocks and soil, is classified as a class I carcinogen by the International Agency for Research on Cancer (IARC)^22^. Up to 21,000 cases of lung cancer-related deaths in the US annually are due to radon, leading the US Centers for Disease Control and Prevention (CDC) to recommend that all homes be tested for radon, but radon testing is not federally mandated as a requirement for a home sale^23^. A meta-analysis containing estimates from 4 pooled studies (incorporating data from 24 individual case-control studies), 1 case-control study and 1 cohort study reported an adjusted excess relative risk of lung cancer of 0.15 (95% CI 0.06–0.25) per 100 becquerels per cubic meter (Bq/m^3^) increase in radon^24^. For homes that have radon levels at or above 4 picocuries per liter (pCi/L), or 150 Bq/m^3^, the Environmental Protection Agency (EPA) recommends reducing radon levels using measures such as **active soil depressurization** and improved ventilation. Because no level of radon exposure is considered safe, the EPA also advises homeowners to consider mitigation for levels between 2 and 4 pCi/L (75–150 Bq/m^3^)^25^.

In 2022, worldwide, approximately 200,000 cases of lung adenocarcinoma, the predominant histologic subtype of lung cancer, were attributed to ambient air pollution, estimated using a population-attributable fraction (PAF) model^8^. Specifically, particulate matter with a diameter of less than 2.5 micrometres (PM_2.5_), found in diesel exhaust or smoke from indoor cooking, can penetrate the alveoli and enter the bloodstream after inhalation. PM_2.5,_ a known cause of lung cancer, promotes tumorigenesis via an influx of macrophages and release of interleukin-1β^26^. Approximately 99% of people worldwide live in areas that exceed World Health Organization (WHO) guidelines on PM_2.5_ (< 5μg m^–3^ annually). A recent multi-country study (6799 lung cancers, including 3615 lung cancer in non-smokers and 26807 controls without lung cancer) reported an association between PM_2.5_ levels and *EGFR*-driven lung cancer incidence, with the relative incidence rates increasing by 0.63-1.82 (per 100,000 population), per 1μg m^–3^ increase of PM_2.5_^27^.

Other environmental particulates associated with lung cancer in non-smokers include asbestos and silica, which are considered Group 1 carcinogens by the IARC^28^. Second hand smoke exposure is also associated with an increased risk of lung cancer among non-smokers. A systematic review of a retrospective observational cohort and 5 case-control studies that included 622469 individuals reported a HR of 1.28 (95% CI: 1.10–1.48) for lung cancer in non-smokers^29^. A secondary analysis of the Global Burden of Disease Study data reported that second hand smoke was responsible for nearly 100,000 deaths worldwide in 2021^30^. Prior radiotherapy to the chest is another risk factor for lung cancer; a retrospective observational study that found 1.74% of 613,746 patients (smoking status unavailable) who received radiotherapy during breast cancer treatment subsequently developed lung cancer^31^.

### Familial and Genetic Risk

Data from a case-control study of 24,380 individuals with lung cancer and 23,399 controls indicate that individuals with a first-degree relative with lung cancer had an odds ratio of 1.51 (95% CI 1.39-1.63) for developing lung cancer compared to those without this family history^32^.

Large scale genome-wide association studies (GWAS) have identifed a pattern of low penetrance germline variants in regions such as 5p15.33 and 3q28 associated with increased risk of lung cancer in non-smokers. These variants involve multiple genes associated with cell cycle regulation, DNA damage, immune response and genomic stability^33^. Singular, highly penetrant germline variants in genes such as *EGFR* and *YAP1* are rare, but have been reported in familial lung cancers from Western and Asian populations, respectively^34,35^. Clonal hematopoiesis (CH), the age-related expansion of a subpopulation of hematopoietic cells with acquired somatic mutations, has been associated with the incidence of many diseases, including lung cancer. In a CH case-control study of 104 cases of incident lung cancers and 343 age-, smoking status- and sex-matched controls, the odds ratio for risk of incident lung cancer was 1.43 (95% CI 1.06-1.94). Although limited by the relatively small number of non-smokers (11 cases and 35 controls) included in the analysis this effect may be independent of smoking history^36^.

Compared with current or former smokers with lung cancer, non-smokers with lung cancer have higher rates of somatic variants and gene rearrangements that activate genes that directly contribute to carcinogenesis and represent potential molecular targets. For example, in a study of 17,712 patients with lung cancer, somatic variants in the *EGFR* gene were identified in 40-60% of lung cancer tumor tissue in non-smokers compared with approximately 10% of smokers with lung cancer^37^. A study of 121 patients with lung cancer younger than age 40 years (73% non-smokers) indicated that 84% carried an actionable oncogenic variant^38^ (Table 2). Rearrangements in the *ALK* and *ROS1* genes are found in 5-14% and 1-2% in tumors of patients with lung cancer, but with higher rates in non-smokers (OR = 3.57 95%CI=2.04-6.25 for *RET* fusions in non-smokers compared to smokers)^39,40^. Other actionable genomic alterations found more frequently in lung cancer biopsy specimens from non-smokers compared with current or former smokers include somatic mutations in *HER2 (ERBB2)*, and gene fusions or rearrangements in *RET, NTRK1/2/3* and *NRG1*^40,41^. A study of non-smoking patients with lung adenocarcinomas (n=160) reported 78%-92% had clinically actionable driver mutations compared with 49.5% in ever smokers (n=299)^11^. A study of 188 lung adenocarcinomas that included 20 non-smokers found that non-smokers had a 10-fold lower tumor mutational burden, defined as the total number of DNA mutations in cancer cells, than tumors from smokers^42^.

#### Screening

To reduce lung cancer mortality, US Preventive Services Task Force (USPSTF) guidelines recommend low-dose CT scanning for individuals aged 50-80 years with a ≥ 20 pack-year history of smoking who currently smoke or quit within the past 15 years^43–46^. However, the USPSTF does not currently recommend screening for lung cancer in non-smokers^47^. In contrast, Taiwan initiated a national lung cancer early detection program in 2022, offering biennial low-dose CT scans to non-smokers (aged 45-74 years for females and 50-74 years for males) with a family history of lung cancer^48^. This screening program was based on results from a study that performed CT screening for 12,011 asymptomatic Taiwanese non-smokers aged 55-75 years with risk factors, such as having a first degree relative with lung cancer, passive smoking exposure, or cooking without ventilation. This study reported that 2.7% with a family history of lung cancer were diagnosed with incident lung cancer, in contrast to 1.6% without a family history (p <0.0001), and 77.4% of lung cancers were diagnosed at Stage I^48^.

#### Clinical Presentation

Among individuals diagnosed with lung cancer, non-smokers have a similar clinical presentation as individuals with a history of tobacco consumption^11^. In a registry of 9876 patients diagnosed with lung cancer in Spain ,1177 (11.9%) were non smokers. Among non smokers, the most common symptoms at diagnosis were cough (34%), dyspnea (29%), pain (28%) and weight loss (19%). The percentage who were asymptomatic (31.5% of all patients) did not differ when stratified by smoking status^49^. In a retrospective series of 539 patients who underwent surgical resection for primary lung cancer at the University of California, Los Angeles, 345 (64%) were asymptomatic and had their tumors discovered incidentally on CT imaging and 143 (41%) were never-smokers^50^.

#### Assessment and Diagnosis

##### Imaging

The recommended imaging study for patients with chest symptoms concerning for lung cancer or with an abnormality detected on chest radiograph, is a contrast-enhanced chest CT to evaluate the primary lesion and assess for nodal involvement and **metastases**.

Brain imaging, preferably MRI, should be performed in all patients diagnosed with lung cancer, as 10-25% have brain metastases at diagnosis^51^. This rate may be higher in patients with genomic alterations such as *EGFR* variants and *ALK* or RET fusions with 52% of 50 patients presenting with brain metastases having an *EGFR* mutation in 1 study^51^. Fluorodeoxyglucose (FDG) Positron emission tomography (PET)-CT should be performed for patients being considered for potentially curative local therapy (such as radiotherapy or surgery) to determine extent of nodal involvement and to exclude metastatic disease^52^.

Patients with indeterminate pulmonary nodules (a focal distinct radiographic density surrounded by lung tissue) should undergo follow-up imaging based on the Fleischner Society guidelines (Supplementary Table 1). More than 95% of indeterminate pulmonary nodules are benign and the probability of malignancy is less than 1% for all nodules smaller than 6 mm (Supplementary Table 1)^53^.

##### Tissue Diagnosis and Molecular Testing

Biopsy of tumor tissue in the lung and/or lymph nodes performed with percutaneous biopsy or bronchoscopic biopsy, often guided by endobronchial ultrasound, confirms the diagnosis and may be useful for staging. Molecular testing should be performed for all non-smokers with lung cancer to aid decision making about use of targeted therapy and immunotherapy^54^. High throughput next generation sequencing (NGS) with DNA and RNA panels is currently recommended to identify genetic variants or rearrangements in genes including *EGFR, ALK, ROS1* and *RET* for consideration of targeted therapies^54^. Characterization of circulating tumor DNA (ctDNA) or “liquid biopsy” is emerging as a test for detecting actionable genomic variants in plasma and may be particularly useful if limited tissue is available for molecular analyses. A study of 171 individuals with lung cancer reported that compared with patients with ctDNA-high status (n = 38), those with a pre-operative ctDNA-negative status (*n* = 18) had improved 5-year overall survival: 100%; 95% CI, 100%–100% vs 48.8%; 95% CI, 34.7%–68.7% (p=0.0024)^55^. Among patients who develop resistance to treatment, re-biopsy of lung cancer or liquid biopsy can reveal mechanisms of resistance such as alterations in genes encoding components of the PI3K-AKT MTOR pathway and alterations in RAS, which can guide subsequent treatment strategies^56^.

#### Staging

Most non-smokers with lung cancer are diagnosed at an advanced stage, typically with unresectable locally advanced disease (stage III) or distant metastases (stage IV). In a retrospective cohort study of 254 non-smoking patients with lung cancer, 62.9% were diagnosed with stage III or IV disease^57^. In another retrospective cohort study of 795 non-smoking patients, 43.4% were diagnosed with Stage IIIB-IV at diagnosis^57^. Early dectection of lung cancer in non-smokers may be difficult due to absence of routine screening in this population and the often non-specific symptoms such as cough and fatigue. However, staging at diagnosis can vary significantly by geography and by use of CT screening. For example, in the targeted low-dose CT screening trial for high-risk never-smokers in Taiwan, 246/257 (95.7%) were diagnosed with stage IA or IB lung cancer, demonstrating the potential effect of screening in shifting diagnosis toward earlier stages^48^.

#### Treatment

Treatment for lung cancer is guided by the stage, histology and molecular status of the tumor, patient performance status, comorbidities, and preferences, and does not differ by smoking status. Treatment options primarily include surgery, radiation therapy (RT), and systemic therapy often given in combination depending on disease stage and often involving multidisciplinary assessment and treatment by surgeons, medical and radiation oncologists, radiologists, pathologists, and nurses to deliver personalized treatment recommendations^65,66^.

#### Early-stage and locally advanced lung cancer

Surgical resection is preferred treatment for patients with anatomically resectable lung cancer (Stage I-III) who are medically eligible for surgery, with follow-up CT screening recommended every 6 months for 2-3 years, and then annually. For patients with stage IB-IIIA and resected *EGFR* variant positive or *ALK* rearranged NSCLC, targeted therapies are effective in both non-smokers and smokers. A phase 3 trial of 682 patients with stage IB-IIIA completely resected *EGFR*-variant NSCLC reported that among patients with stage II-IIIA disease randomized to 3 years of treatment with the EGFR Tyrosine Kinase Inhibitor (TKI) osimertinib 80 mg once-daily or placebo, 4-year disease-free survival (DFS) was 70% in the osimertinib group vs 29% in the placebo group, with DFS HR of 0.23 (95% CI, 0.18 to 0.30) at a median follow-up of 44.2 months (osimertinib) and 19.6 months (placebo)^58^.The Hazard Ratio (HR) for death or recurrence at 2 years was 0.23 [95% CI, 0.15-0.34] in non-smokers (n=488), and 0.10 [95% CI, 0.04-0.22] in those who had smoked (n=194)^59^. A follow-up study of this cohort of patients with stage IB to IIIA disease reported a 5-year overall survival (OS) of 85% in the osimertinib group vs 73% in the placebo group (overall HR for death, 0.49; 95% CI, 0.33 to 0.73; P<0.001)^60^. In a trial of 257 patients with stage IB, II or III resected *ALK* rearrangement positive NSCLC, after 2 years of adjuvant TKI (alectinib) or platinum-based chemotherapy, among patients with stage II or IIIA lung cancer, DFS at 2 years was 93.8% in the alectinib group and 63.0% in the chemotherapy group (hazard ratio for disease recurrence or death, 0.24; 95% CI, 0.13 to 0.45; P<0.001)^61^.

A trial that included 216 patients unresectable *EGFR*-mutated stage III NSCLC without progression during or after chemoradiotherapy reported improved median PFS with adjuvant osimertinib vs placebo (39.1 months vs 5.6 months), with a HR for disease progression or death of 0.22 (95% CI 0.14-0.34) in non-smokers (n=102), and 0.26 (95% CI 0.14-0.48) in smokers (n=41)^62^.

Immunotherapy with PD-1 or PD-L1 inhibitors are commonly prescribed neoadjuvant or adjuvant treatments for patients with early-stage lung cancer. However, their role for treating non-smokers with lung cancer is less clear due to a lower likelihood of response to immunotherapy, particularly in the context of *EGFR* gene variants or *ALK* gene rearrangements. A pooled analysis of 3 randomized trials that included treating individuals with nivolumab (n=292), pembrolizumab (n=691), or atezolimuab (n=144),vs docetaxel (n=776) reported that single-agent immunotherapy did not improve overall survival in *EGFR*-variant NSCLC, regardless of smoking status (HR=1.05, 95% CI: 0.70-1.55)^63^. Additionally, patients with *EGFR* gene variants and *ALK* gene rearrangements have often been excluded from peri-operative and adjuvant studies of immunotherapy for patients with lung cancer; few non-smokers have therefore been included in these studies^64,65^. In one trial, 797 patients with stage II, IIIA, or IIIB NSCLC were randomized to the PD-1 inhibitor pembrolizumab 200mg (n=397), or placebo (n=400) given with chemotherapy prior to surgery and for 12 months after surgery vs pre-operative chemotherapy alone.^66^ OS in all patients at 36-months was 71% (95% CI 66–76) in the pembrolizumab group and 64% (95% CI 58–69) in the placebo group (hazard ratio 0.72 [95% CI 0.56–0.93])^66^. The greatest benefit for perioperative pembrolizumab were seen in current smokers (HR for OS 0.59 (95% CI 0.38-0.93), with less benefit seen in non-smokers (HR 1.00 (95% CI 0.41-2.46)^67^.

### Unresectable locally advanced or Metastatic Lung Cancer

For patients with advanced stage lung cancer, treatment typically consists of a combination of chemotherapy, immunotherapy or targeted therapies.Treatment with targeted agents have shown benefits over chemotherapy for indviduals who have *EGFR* variants, *ALK* rearrangements and *RET* rearrangements. A trial that randomized 556 patients with advanced stage *EGFR*-variant lung cancer to osimertinib or a first generation oral EGFR-tyrosine kinase inhibitor reported the hazard ratio for disease progression or death was significant in both those who smoked (n=199), HR 0.48 for osimertinib vs EGFR-tyrosine kinase inhibitor, (95% CI 0.34-0.68) and non-smokers (n=357) HR 0.45, (95% CI 0.34-0.59)^67^. Overall survival was also improved with firstline osimertinib (38.6 months vs 31.8 months, HR 0.80)^68^. A post hoc analysis of 5-year outcomes of the CROWN study, in which 296 patients with advanced ALK-positive non-small cell lung cancer were randomized to lorlatanib (an ALK inhibitor) or crizotinib (a TKI), reported improved outcomes with lorlatinib.^69^ In the lorlatinib group, median PFS was not reached (NR [95% CI, 64.3 to NR]) vs 9.1 months (95% CI, 7.4 to 10.9) in the crizotinib group ([HR], 0.19 [95% CI, 0.13 to 0.27]); 5-year PFS was 60% (95% CI, 51 to 68) in the lorlatinib group vs 8% (95% CI, 3 to 14) in the crizotinib group^69^. Other FDA-approved oncogene driven therapies such as repotrectinib or taletrectinib for ROS-1 positive tumors have demonstrated higher response rates and durations of response than seen historically with chemotherapy in both smokers and non-smokers with objective responses rates, defined as the percentage of patients with a partial or complete response to therapy, seen in 89% of individuals (n=152) for taletrectinib and 79% of individuals with repotrectinib (n=71)^70,71^ (Table 2).

For lung cancers without actionable genomic alterations that can be treated with targeted therapy, chemotherapy combined with immunotherapy can be considered^72,73^. However, single-agent immunotherapy with inhibitors of PD-1 or PD-L1 such as pembrolizumab have limited efficacy in non-smokers, particularly with *EGFR* or *ALK* alterations, and should not be used for non-smoking patients with these variants and unresectable locally advanced or metastatic lung cancer^74^.

Local therapy, such as radiotherapy or surgery may be used to treat sites of metastatic disease. For brain metastases, stereotactic radiosurgery can be used in preference to whole brain radiotherapy to minimize CNS toxicity including cognitive effects^75^. TKIs with excellent CNS penetration such as the EGFR inhibitor, osimertinib, or the ALK inhibitor, lorlatinib, may be used instead of radiotherapy for brain metastasis in many patients^62^. Stereotactic ablative radiotherapy (SABR) is recommended for oligometastatic disease in conjunction with systemic treatment due to its ability to deliver more targeted radiotherapy to a region^76^. In an open-label phase 2 trial of 99 patients with a controlled primary tumor and 1 to 5 metastases, median OS was 41 months (95% CI 26-not reached) in patients randomized to SABR vs 28 months (95% CI 19-33) with standard care^77^. However, adverse events (grade 2 or higher) occurred in 19/99 (29%) of patients in the SABR group vs 3(9%) of 33 in the standard care group (p=0·026), and treatment-related deaths occurred in 3/66 (4·5%) patients after SABR, compared with 0 in the standard care group.^77^

#### Prognosis

Lung cancer survival primarily depends on stage at diagnosis (5-year survival rate of 65% for Stage I vs < 10% for stage IV), performance status and presence of actionable genetic alterations for treatment^78^. A prospective cohort study of 5594 patients with NSCLC (61.8% lung adenocarcinoma) reported a median OS of 58.9 months (95% CI 51.9-67.4) for 795 non-smokers (55.8% Stage 1A -IIIA, 43.4% Stage IIIB-IV), a median OS of 51.2 months (95% CI 47.7-54.6) for 3308 former smokers (68.9% Stage 1A-IIIA, 30.1% Stage IIIB -IV) and a median OS of 34 months (95% CI 29.1-42.3) for 1491current smokers (63.6% Stage 1A-IIIA and 35.9% Stage IIIB-IV)^57^.

Significant improvements in PFS and OS among patients with lung cancer have occurred with use of targeted therapies directed at oncogenic drivers^52,79,80^. A population-based study using the Surveillance, Epidemiology, and End Results (SEER) data reported that 2-year lung cancer-specific survival improved from 26% for patients diagnosed in 2001 to 35% in 2014, a change largely attributable to the advent of targeted therapies. A French study reported that median OS among patients diagnosed with lung adenocarcinoma increased from 8.5 months in 2000 (n=1684) to 20.7 months in 2020 (n=5015), although no information on smoking status was provided^81^. The median OS for advanced *EGFR* variant positive lung cancer treated with EGFR inhibitors is currently 38.6 months and the median OS for patients with advanced *ALK* positive NSCLC treated with ALK inhibitors exceeds 5 years among smokers and non-smokers (Table 2)^68,82^.

### Practical Considerations

Individuals with lung cancer may face stigma due to the disease's association with smoking, which can lead to feelings of isolation^74^. Patient education and psychosocial support for non-smokers with lung cancer enhances well-being and may improve outcomes through greater adherence to treatment as found in a systematic review of 18 cohorts^73^. A trial of 151 patients with metastatic lung cancer randomized to early palliative care (n=77 with 18 non-smokers) vs standard of care (n=74 with 16 non-smokers) found that early palliative care led to significant improvement in quality of life, reduced depressive symptoms and improved median survival (11.6 months vs 8. 9 months, P=0.02)^83^. Many support and advocacy groups exist, including those focusing on specific molecular subtypes of lung cancer (Table 3). Proactive follow-up of respiratory symptoms with CT imaging, regardless of a patient’s smoking history, could lead to earlier-stage lung-cancer diagnosis, a message echoed by awareness campaigns such as “See Through the Symptoms” and “All You Need Is Lungs”^9^.

### Future Directions

Ongoing trials, such as the Female Asian Nonsmoker Screening Study (FANSS) study in the US are evaluating potential benefits of screening for lung cancer among high-risk groups of non-smokers^84^. In addition, studies of biomarker-based early detection approaches in blood (such as multi-cancer early detection tests and proteomic approaches) are in progress to determine if lung cancers can be detected at an early and more curable stage.

### Limitations

This review has several limitations. First, smoking history is often not included in many databases, cancer registries and clinical trials, making it difficult to accurately determine the incidence and prevalence of lung cancer in non-smokers and to evaluate treatment outcomes based on smoking status. Second, accurate quantification of environmental exposures, such as air pollution, is challenging. Third, the quality of the evidence was not formally evaluated. Fourth, some articles may have been missed.

## Conclusions

Lung Cancer in non-smokers accounts for 15-20% of the lung cancer cases worldwide. Among patients with lung cancer, non-smokers are more likely to have genomic alterations such as *EGFR* variants or *ALK* gene rearrangements, and these patients have improved survival when treated with tyrosine kinase inhibitors, compared with chemotherapy.

## Supplementary Material

Supplementary table

## Figures and Tables

**Table 1:** The principal differences between lung cancer in people who have never smoked (LCINS) and lung cancer in people who have smoked (LCIPS). Mb; Megabase. SBS4: The Catalogue of Somatic Mutations in Cancer (COSMIC) mutational profile associated with smoking, characterised by a transcriptional bias for C > A mutations.

Characteristic	Lung cancer in people who have never smoked	Lung cancer in people who have smoked
Median age at diagnosis	67 years^21^	70 years^21^
Proportion of new lung cancer diagnoses worldwide	15%^4,6^	85%^4,6^
Absolute Incidence	0.16 per 1000 person years^85^	2.99 per 1000 person years
Lung Adenocarcinoma Risk factors (% of total cases the risk factor is thought to contribute to)	Targetable Genetic Alterations (70-80%)^5,11,86^ Second hand smoke (15%) Occupational exposures (13%) Radon (9%) Air pollution (8%) Household cooking and heating (4%) Prior Radiotherapy (0.3% of those exposed) Female sex (Up to 80% in some cohorts)	Smoking (100%) Targetable Genetic Alterations (49.5%)^11^ Second hand smoke (15%)* Occupational exposures (10%)* Radon (9%)* Air pollution (8%)* Prior Radiotherapy (4% of those exposed) Female sex (40%)
Screening recommendations	No national screening programs other than Taiwan^87^	US: Annual low dose CT screen for those aged 50-80 with ≥20 pack year history and smoked within last 15 years^44^
Sex Difference	19% of females with lung cancer 9% of males with lung cancer^21^	81% of females with lung cancer 91% of males with lung cancer
Survival Benefit with Immunotherapy	Hazard Ratio (HR)= 0.87, Confidence Interval (CI) 0.74-1.74^74^	HR 0.74, CI 0.69-0.81
Lung Adenocarcinoma Frequency of oncogenic drivers (see Table 2)	78-92%^11^	49.5%^88^
Tumor Mutation Burden	0-3 mutations / Mb^89^	0-30 mutations / Mb^89^
Genomic signature	None	Smoking signature (SBS4)^90^
Histology	Lung Adenocarcinoma (60-80%), Small Cell (8%), Squamous Cell (17%)^91^	Lung Adenocarcinoma (24%), Small Cell (17%), Squamous Cell (41%)^91^

* Effects are additive in those who have smoked.

**Table 2:** Driver mutation frequency and current availability of targeted FDA approved drugs. Frequency data from whole exome sequencing profiling of 160 people with lung cancer. Smoking values are weighted averages among the current and former smoker categories^11^. NR = Not Reached meaning data too immature to calculate data from.

Oncogene	Mutation Frequency in LCINS	Mutation Frequency in LCIPS	Targetable alteration	FDA Approved Drugs (Target alteration)	Year of First approval	Median Overall Survival (months)	Median Progression Free Survival (months)	Delivery route
*EGFR*	43%	11%	1) Exon 19 Deletion or Exon 21 L858R	Afatanib (1,2)	2018	27.9	11	Oral
				Amivantamab (3)	2024	NR	11.4	Intravenous
				Dacomitinib (1,2)	2018	34.1	14.7	Oral
				Erlotinib (1,2)	2016	84.2	10	Oral
				Gefitinib (1,2)	2003	27	9.2	Oral
			2) S768I, L861Q, and/or G719X	Osimertinib (1,2)	2015	38.6	18.9	Oral
			*3)* Exon 20 Insertion Mutation	Amivantamab + chemotherapy	2024	38.9	20.6	Oral
*ALK*	12%	2%	Rearrangement	Alectinib	2015	NR	34.8	Oral
				Brigatinib	2017	NR	16.7	Oral
				Ceritinib	2014	51.3	16.6	Oral
				Crizotinib	2015	NR	10.9	Oral
				Ensartinib	2024	NR	25.8	Oral
				Lorlatinib	2018	NR	NR	Oral
*KRAS*	9.10%	29%	G12C	Adagrasib	2022	NR	7.4	Oral
				Sotorasib	2021	12.5	6.3	Oral
*ROS1*	3.22%	1.11%	Rearrangement	Entrectinib	2019	47.8	15.7	Oral
				Crizotinib	2011	NR	10.9	Oral
				Repotrectinib	2023	NR	35.7	Oral
				Taletrectinib	2025	NR	45.6	Oral
*ERBB2* *(HER2)*	2.22%	1.34%	Mutation	Fam-Trastuzumab Deruxtecan	2022	17.8	8.2	Intravenous
*RET*	2%	0.5%	Rearrangement	Pralsetinib	2022	21.2	10.7	Oral
				Selpercatinib	2022	NR	24.8	Oral
*BRAF*	1.83%	4.12%	V600E	Encorafenib/ Binimetinib	2018	33.6	14.9	Oral
				Dabrafenib/ Trametinib	2013	25.9	11.1	Oral
*MET*	1.5%	2.1%	Exon 14 Skipping	Capmatinib	2020	20.8	10.8	Oral
				Tepotinib	2021	29.7	15.9	Oral
*NRG1*	0.18%	0.08%	Gene Fusion	Zenocutuzumab	2024	Not reported	6.8	Intravenous
*NTRK*	~0.17%*		Gene Fusion	Entrectinib	2019	41.5	28	Oral
				Repotrectinib	2024	NR	NR	Oral

* = reliable data by smoking status not available.

**Table 3:** List of lung cancer support groups available to patients, clinicians and scientists for advocacy, support and funding.

Support Group	Role/Focus
ALK-Positive	Support and advocacy for patients with *ALK*-positive lung cancer, focusing on research and education.
BRAF-Bombers	Dedicated to supporting and educating patients with *BRAF*-mutated lung cancer.
EGFR-Resisters	Support and advocacy group for patients with *EGFR*-mutated lung cancer, focusing on treatment resistance.
Exon20 Group	Specialises in supporting patients with *EGFR* Exon 20 insertion mutations, advocating for research and treatments.
KRAS Kickers	Supports patients with *KRAS*-mutated lung cancer, promoting research and sharing treatment information.
LUNGevity Foundation	Offers support, education, and funding for lung cancer research, focusing on early detection and treatment.
MET crusaders	Advocacy for those affected by lung cancer with a *MET* mutation
RET positive	Supports patient with forms of *RET* positive cancers
ROS1ders	Seeks to improve outcomes for those with *ROS1* positive cancers

## Box

QUESTION: “How common is lung cancer in non-smokers and what are known risk factors?

ANSWER: “Worldwide, lung cancer in non-smokers accounts for 15-20% of all lung cancers. Exposure to passive smoking, radon, air pollution and asbestos, and having a first-degree relative with lung cancer are common risk factors for lung cancer in non-smokers.”

QUESTION: Among patients with lung cancer, which genetic variants are more common among non-smokers compared with former or current smokers?

ANSWER: Non-smokers with lung cancer have higher rates of genomic alterations such as *EGFR* variants or *ALK* gene rearrangements that directly contribute to carcinogenesis and represent potential molecular targets. Among patients with these genetic alterations, use of tyrosine kinase inhibitors (osimertinib and lorlatinib) is associated with improved survival.

Question: What are the recommendations about screening for lung cancer in non-smokers?

ANSWER: The US Preventive Services Task Force (USPSTF) does not recommend screening non-smokers for lung cancer with low-dose CT scans. In contrast, Taiwan initiated a national lung cancer early detection program in 2022 that offers biennial low-dose CT scans to non-smokers with a family history of lung cancer.
