# Supplementary material for: Lung cancer In non-smokers
Source: JAMA. Author manuscript; Available in PMC 2025 Nov 18. (PMC7618360; doi:10.1001/jama.2025.17695)
Supplement: Supplementary table [file EMS210578-supplement-Supplementary_table.docx]

| **Nodule Type** | **Size /Component** | **Considerations** | **Recommend Follow-up** |
| --- | --- | --- | --- |
| Solid Nodule | <6mm | **Low risk:** No routine follow-up  **High risk:** Consider CT at 12 months | Low risk: No routine follow-up  High-risk: CT follow-up at 12 months |
| Solid Nodule | 6-8mm | Not stratified by risk in this size range | CT scan at 6–12 months; if unchanged, an additional CT at 18–24 months |
| Solid Nodule | >8mm | Higher malignancy risk | Evaluation at 3 months  with CT, PET-CT, or biopsy |
| Ground-Glass Nodule | < 6mm | Generally low concern | No routine follow-up  required |
| Ground-Glass Nodule | > 6mm | Persistence needs to be assessed | Initial CT at 6–12 months; if stable, further imaging  at 2-year intervals for up to 5 years |
| Part-Solid Nodule | Solid component and  < 6mm | Lower risk if stable | CT at 3–6 months; if unchanged, annual CT  scans for 5 years |
| Part-Solid Nodule | Solid component ≥ 6  mm | Considered highly suspicious for malignancy | More immediate evaluation recommended, such as biopsy or surgical  resection |

**Supplementary Table 1**. The management of indeterminate pulmonary nodules based on the

Fleischner Society guidelines. Follow-up differs for those deemed to be low or high risk

(assessing risk factors such as smoking status and age) but it is ultimately judged by the

clinician responsible for the patients care^92^.
